# Inequalities in the Challenges Affecting Children and their Families during COVID-19 with School Closures and Reopenings: A Qualitative Study

**DOI:** 10.1093/phe/phac030

**Published:** 2022-12-14

**Authors:** Ilaria Galasso, Gemma Watts

**Affiliations:** University College Dublin; University College Dublin

## Abstract

School closure is one of the most debated measures undertaken to contain the spread of the Coronavirus disease (COVID-19) pandemic. The pandemic has devastating health and socio-economic effects and must be contained, but schools play a vital role in present and future well-being, capabilities and health of children. We examine the detrimental consequences of both the closure and reopening of schools, by focusing on inequalities in the challenges affecting children and their families. This paper is grounded on Irish and Italian data from a multi-national longitudinal qualitative interview study. Research participants articulated a variety of issues and challenges that highlight inequalities in access to education during school closures, in the supportiveness of home setting, and in school preparedness to reopen, often mirroring or exacerbating pre-existing inequalities. The reported unequal lived experiences indicate that some harms are actionable, and already suggest some potential harm mitigation strategies. We conclude by advocating for enhanced public consultation to help mitigate the consequences of public dilemmas in general, and to help detect and tackle inadequacies and inequalities for school children through and beyond the pandemic, by learning from the experience of the concerned actors.

## Introduction

Children may not have been the greatest victim of Coronavirus disease (COVID-19) but may suffer the biggest detrimental effect because of the pandemic. Preventative measures have been implemented by governments globally, to contain and mitigate the number of cases and indeed deaths from the COVID-19 pandemic. One of these preventative measures was the closure of schools nationally: as of April 2020, 188 countries worldwide had implemented these closures (United Nations, 2020). The time period for these school closures varied from country to country and was determined at national level.

In this paper, we focus on school closures and reopening throughout the year 2020 in Ireland and Italy, as illustrative examples. In Ireland, closure of all schools was ordered from 12 March 2020 (MerrionStreet.ie, 2020). In Italy, all schools in the Northern region (where the first clusters occurred) closed on 23 February 2020, and in the whole country from 4 March 2020 (DECRETO-LEGGE 23 febbraio, 2020). In both countries, schools did not reopen until the beginning of the new school year (end August–early September at schools’ discretion in Ireland; between 7 and 24 September, depending on the region, in Italy). In Ireland, schools remained open for the remainder of 2020. In Italy, from 16 October 2020 regional closures started (ORDINANZA n. 79 del 15 ottobre, 2020), with new periodical or partial closures depending on the evolving pandemic situation in specific regions as evaluated by the Ministry of Health (DCPM 3 novembre, 2020).

This paper, grounded on lived experiences of concerned public as reported through qualitative interviews in Ireland and Italy in critical timeframes (April–May 2020, when schools were completely closed, and October–November 2022, when schools had recently reopened while new closures were under political consideration), examines inequalities in the challenges affecting children and their families related to primarily the closure of schools, but also their subsequent reopening, as this too presented challenges that will be discussed later. Through our analysis of data, we discuss the two options faced by governments–closing schools as a preventative measure with the aim to mitigate the spread of the virus versus not closing schools for securing education continuity during a worldwide pandemic—which we see as a ‘public health dilemma’ and we will discuss in more detail later. We argue that consultation of the concerned public plays a key role in achieving a better understanding of and giving visibility to the unequal challenges related to institutional interventions and that it can provide informative guidance to mitigate detrimental and unequal consequences of this and other public health dilemmas.

This paper is developed as part of the multi-national, qualitative, longitudinal study ‘Solidarity in Times of Pandemic’ (SolPan) and is grounded on interviews conducted in Ireland and Italy. Our qualitative interviews, by collecting lived experiences and whole first-hand stories, allow a deep and comprehensive analysis of the different (and unequal) challenges at stake in both scenarios (schools open and closed). In particular, this qualitative research brings original insights on inequalities: while concerns and requests brought to the public arena mostly involve situation of discontent, our data also includes situations of acceptance and possibly even of satisfaction—situations in which interview participants reported efficient assistance from schools and/or social and domestic facilitations, which they associated to minor challenges, or no challenges or even benefits in relation to school closure and with reopening, respectively.

Although we acknowledge that in different countries, issues and unequal challenges related to school closure and reopening may be dissimilar, and of differing scale, depending on the social, cultural, political, educational and pandemic context; we believe—considering the global scale of the many existing studies on the consequences of education disruption (as displayed in the next subsection)—that our findings may have relevance in all countries where school closure has been implemented as a pandemic containment measure. Moreover, we expand our argument to state that consultation with the concerned public can helpfully mitigate the consequence of public health dilemmas in any context.

### The Multi-Edged Detrimental and Unequal Effects of School Closure

According to the United Nations Children’s Fund (UNICEF), between March 2020 and February 2021, 214 million children globally have missed more than three-quarters of their in-person learning due to the COVID-19 pandemic restrictions, and more than 168 million schools have been completely closed for almost an entire year (UNICEF, 2021). The United Nations defined it as ‘a generational catastrophe’ (United Nations, 2020: 3), which has disproportionately affected the most vulnerable and marginalized children and youth, and warned that almost 24 million children and young adults may never go back to school due to the pandemic’s economic impact.

Given the (different dependent on age and individual context, but always primary) role that school has in children’s (and parents’) daily routine, as well as educational and social life, long-term school closure is expected to have long-lasting devastating effects. School closure forces the educational and social interaction setting to be the home, and is therefore reliant on and affected by that child’s home environment (OECD, 2020). This has exacerbated existing inequalities as it has been further detrimental for the already more disadvantaged children and for the lower income families (Marmot et al., 2020; United Nations, 2020; Engzell et al., 2021; UNESCO, 2022).

The obvious consequences of school closures relate to children’s learning and education, but education disruption is expected to affect several other important aspects of life, ranging from freedom to health. Education has been argued to play a central role in terms of fair equality of opportunities (Rawls, 2001), in terms of capabilities (Walker and Unterhalter, 2007) of what people are able to do and to be (Sen, 1979), and consequently in terms of freedom as the substantial opportunity to achieve what is wanted and valued (Sen, 1999, 2002). Education has also been argued as a key factor in terms of health (Daniels, 2008; Marmot et al., 2020). In this respect, by analyzing existing data and referring to several studies evidencing a correlation between education level and life expectancy, Christakis et al. estimated that the educational disruption due to school closure during the COVID-19 pandemic may cause 5.53 million years of life lost globally: more, according to the authors, than would have been lost due to COVID-19 mortality (Christakis et al., 2020).

Moreover, school closure does not have implications only in terms of education disruption, but importantly also on many physical, mental and social aspects of children’s well-being: as many studies report (Marmot et al., 2020; OECD, 2020; UNESCO, 2022), it increases children’s social isolation, risk of poor nutrition, potential exposure to violence and exploitation; it is expected to cause stress to parents, and even work and wage loss, with further economic and emotional consequences for children themselves. Therefore, inequalities in education are expected in the long term to translate into broader social, economic and health inequalities, and an exacerbation of education inequalities will correspond to an exacerbation of these other inequalities.

In this paper we report, illuminate and disentangle some *specific* inequalities exacerbating the effect of school closure and reopening in the context of the pandemic, with the aim—depending on the case and on the actionability of the various inequalities—to help decisions around the public health dilemma of school opening and closing by including specific inequalities in the balance, or to promote their mitigation.

### A Public Health Dilemma

In the previous section, we argued that society has a moral duty to ensure access to education for school aged children, because of the role education plays in terms of capability and equality of opportunity for the recipients. Here we argue that society has the likewise important moral duty to promote good health, for the same reason: the right to health is also defended in terms of equality of opportunities and of capabilities (see Daniels, 1981, 1985, 2008, 2010, 2017). In the case of COVID-19, to also add to this obligation, given the emotional and physical suffering often generated by COVID-19 infection, its prevention is also supported by the principle of beneficence and the ethical obligation to prevent harm (Buchanan, 1984). Moreover, sickness and death have serious disruptive consequences at many levels not only on the person incurring them: the emotional, financial, logistical costs of long-term illness and premature death often weigh heavy on family members, and may compromise their opportunities and capabilities to the further detriment of social equity (Golics et al., 2013). Once again, the highest multi-level cost is for children, especially in the millions of cases in which they were orphaned by COVID-19 (Flaxman and Hills, 2022; Unwin et al., 2022). Against this background, we argue that society has a moral duty both not to interrupt children’s education and to prevent the spread of COVID-19.

When an agent is required to do one of two actions (but not both) and whatever they do is morally wrong, this is defined as a moral dilemma (McConnell, 2018). Similarly, we argue that when institutions are required to implement one of two public health interventions (but not both), and whatever they do is morally wrong, this is a public health dilemma. As COVID-19 is an infectious disease, one of the initial political reactions in the heat of the outbreak of the pandemic—later in part retracted or at least problematized (see Fukumoto et al., 2021; Monod et al., 2021)—was to identify schools as a major site of contagion (see MerrionStreet.ie, 2020; DECRETO-LEGGE 23 febbraio 2020, regarding this governmental position in Ireland and Italy, respectively). We argue that under this perspective the case of school closures or reopening in the context of the COVID-19 pandemic is an example of a public health dilemma: as discussed in the previous sections, keeping schools closed has detrimental effects on children’s education and well-being, and may crucially and irreversibly affect life opportunities and capabilities especially of children already exposed to inequalities, by exacerbating inequities. On the other hand, policymakers feared that keeping schools open could facilitate—although it is controversial to what extent, as we discuss in this section—the spread of a pandemic causing debilitating long-term illness and in some cases death to the cost of those who get sick and of their families. As discussed in this and in the previous section, in any moral framework defending the principle of beneficence and/or the principle of fairness, neither of these two scenarios is acceptable.

Both promoting children’s well-being and education, and protecting the health of the population—also given the impact that both have on other essential aspects of human life—are thence examples of what the philosopher Lisa Tessman calls ‘non-negotiable moral requirements’ (Tessman, 2015). When two non-negotiable moral requirements are in conflict with one another, neither of them can override the other: in this case we have a *genuine* moral dilemma (a genuine public health dilemma), and either option have morally unacceptable consequences (Sinnot-Armstrong, 1988). Against this background, we argue that the choice of whether to keep schools closed or open in the context of the COVID-19 pandemic is a genuine public health dilemma—or at least it was perceived as such whenever schools were assumed as a major site of contagion.

It is crucial to remark, as matter of fact, that while one side of this dilemma (that school closure causes detrimental and unequal educational and well-being disruption) is quite uncontroversial (Marmot et al., 2020; United Nations, 2020; Engzell et al., 2021); the other side (that schools cause significant harm to the health of the population) is actually the object of major debates, to the extent that it has been called ‘the single biggest issue dividing academics’ (Rigby et al., 2021). Prominent public health experts have argued that schools give a significant contribution to the spread of the COVID-19 pandemic (Auger et al., 2020; Flaxman et al., 2020; Goldstein et al., 2020; Haug et al., 2020;Gurdasani et al., 2021; Hyde, 2021). Other likewise renowned public health experts have claimed that, especially if adequate safety measures are adopted, schools are unlikely to have a major impact on the spread of the pandemic (ECDC, 2020; Ludvigsson, 2020; CDC, 2021); or that, in any case, the social and health damage caused by school closure is more detrimental than the pandemic itself (Christakis et al., 2020; Donohue and Miller, 2020; Pemberton, 2020). Even the several studies conducted *a posteriori* have not reached a final answer in this regard. Interestingly enough however, some of these studies actually observed that especially when other measures were in place, and particularly in relation to primary schools, little significant increase in COVID-19 cases is detectable in correlation to and in the context of school in person activities (see for example, Bonaccorsi et al., 2021; Fukumoto et al., 2021; Walsh et al., 2021; Godøy et al., 2022). Studies also reported that children are less susceptible than adults to COVID-19 infection and, if infected, are significantly less likely than adults to infect others (see Monod et al., 2021; Kraaijeveld et al., 2022).

If—or when—keeping schools open only corresponds to a slightly increased risk of COVID-19 spreading among the population, then there is no conflict or dilemma at all: schools can just be kept open—as political authorities actually did while regulating closures and reopenings based on the evolvement of local pandemic situations—without causing morally wrong consequences. As a matter of fact, philosophers engaged in the framework of ethics of risk, generally agree that to refrain from any action that slightly increases the risk of harming other people would correspond to refraining from virtually every action, and that it would not be ‘socially viable’ (Hansson, 2013). Importantly, within this framework it is still an open issue how to determine which risks are morally acceptable. In the case of COVID-19, however, the initial uncertainty was so huge that the strictest lockdown measures that were implemented in some countries actually required people to literally refrain from virtually every action (Mathieu et al., 2020). This approach also reverberated on decisions about school closure: in certain phases, the risk of contagion was (deemed) so high that keeping them open or closed to protect the health of the pollution was (treated as) a true genuine dilemma.

In this paper we focus on the specific phases in which keeping schools open and protecting the health of the population was a conflict and a public health dilemma, or at least it was managed as such with the (lack of) data that political authorities could avail of, as was the case every time and everywhere schools have been closed as a containment measure during this time period.

## Methods

The SolPan consortium formed in March 2020 and led by Barbara Prainsack, Katharina Kieslich and Wanda Spahl, University of Vienna, conducted a series of longitudinal in-depth qualitative interviews in nine European countries (see Zimmermann et al., 2022). The aim was to explore how and why people responded to COVID-19 and ensuing containment measures, such as school closures and reopenings.

For this paper, we grounded on the interviews conducted in Ireland and in Italy. The interviews were conducted in two rounds: the first round (T1) in April–May 2020 (24 April—5 May in Ireland, 17 April—3 May in Italy), when in both countries lockdown measures had been first implemented and schools were closed, and the second (T2) in October–November 2020 (6 October–5 November in Ireland; 13–31 October Italy), when some containment measures had been lifted and schools had reopened, but more restrictions and new school closures were potentially on the horizon (in two Italian regions, schools closed again while our interviews were still running). In T1, 32 interviews were conducted in Ireland and 33 in Italy. Of these, 25 participants in Ireland and 29 in Italy agreed to be interviewed again in T2. Interviews ranged from 30 to 90 min, and were conducted by telephone or video call in the language of the participant’s country. Recruitment was through convenience sampling, snowballing and online consortium owned websites and social media accounts. Information on the study was made available to potential participants in advance with a further detailed information leaflet circulated prior to the interview. Participants’ consent was gained at the start of each interview. All interviews were transcribed and anonymized. Abbreviated interview codes are included here to indicate the participant’s country of residence (IE for Ireland, IT for Italy) and the time period (T1 or T2). The software programme NVivo was used for the Irish data and Atlas.ti for the Italian data, the interviews were coded with an inductively-generated coding scheme developed by the SolPan Consortium (SolPan Consortium, 2021a) following a grounded theory approach (Charmaz, 2006). The coding of the Irish data was completed and checked by three researchers and the Italian data by two to ensure validity, consistency and interoperability. One of the authors (Galasso) was involved in the data coding for Ireland and Italy and conducted interviews in both countries. The study received ethical approval from University College Dublin (HS-E-20-70-Galasso) and the University of Vienna (00544).

Demographic questions were used to ensure a broad range of perspectives with participants being of different ages, gender, financial situation, family situation living conditions and geographical area (see Tables 1 and 2). The study only included adult participants, so the perceptions of children were not captured directly. However, the SolPan participants with children in the household (nine in Ireland, six of them participated also in T2; 11 in Italy, 10 of them participated also in T2) were affected in and through their children, and all the participants, as part of the society, were concerned with the implications of governmental decisions around schools. The interviews followed the same interview guide, developed by the SolPan Consortium (SolPan Consortium, 2021b). For T1, there were no specific questions focusing on schools or children, however, participants with child-caring responsibilities shared their experience about school closures. In T2 a question on schools was included.

**Table 1.** Self-reported demographic characteristics of participants by country (T1)

Category	IT (*n* = 33)		IE (*n* = 32)	
**Age**				
18–30	3	9%	5	16%
31–45	15	45%	13	40%
46–60	8	24%	8	25%
61–70	3	9%	2	6%
70+	4	12%	4	12%
**Gender**				
Female	22	67%	20	62%
Male	11	33%	12	37%
Other	0	0%	0	0%
**Household**				
Single	7	21%	9	28%
Couple	8	24%	11	34%
Living with child/children under 12	6	18%	5	16%
Living with child/children 12+	5	15%	6	19%
Other	7	21%	1	3%
**Rural/Urban**				
Big town (e.g. capital, +500k)	14	42%	17	53%
Medium/small town	11	33%	10	31%
Rural (e.g. village)	8	24%	5	16%
**Employment status**				
Employed (long-term contract)	10	30%	16	50%
Self-employed	9	27%	4	12%
Employed (short-term/precarious contract)	3	9%	2	6%
Unemployed	2	6%	2	6%
Retired	3	9%	4	12%
Other	6	18%	4	12%
**Education level**				
Less than 10 years	2	6%	2	6%
10–14 years (e.g. highschool diploma)	17	52%	3	9%
Higher education	14	42%	27	84%
**Household net income** (prior to Corona), net income:				
Up to 1400€(1200GBP)/month	5	15%	3	9%
1401(1201)–3000€(2600GBP)/month	22	67%	9	28%
More than 3000€(2600GBP)/month	6	18%	20	62%

**Table 2.** Self-reported demographic characteristics of participants by country (T2)

Category	IT (*n* = 29)		IE (*n* = 25)	
**Age**				
18–30	3	10%	1	4%
31–45	11	38%	12	48%
46–60	8	28%	6	24%
61–70	3	10%	2	8%
70+	4	14%	4	16%
**Gender**				
Female	21	72%	17	68%
Male	8	28%	8	32%
Other	0	0%	0	0%
**Household**				
Single	6	21%	6	24%
Couple	8	28%	11	44%
Living with child/children under 12	5	17%	4	16%
Living with child/children 12+	4	14%	4	16%
other	6	21%	0	0%
**Rural/urban**				
Big town (e.g. capital, +500k)	12	41%	12	48%
Medium/small town	9	31%	9	36%
Rural (e.g. village)	8	28%	4	16%
**Employment status**				
Employed (long-term contract)	9	31%	13	52%
Self-employed	8	28%	4	16%
Employed (short-term/precarious contract)	3	10%	2	8%
Unemployed	2	7%	1	4%
Retired	3	10%	4	16%
other	4	14%	1	4%
**Education level**				
Less than 10 years	2	7%	2	8%
10–14 years (e.g. high school diploma)	16	55%	2	8%
Higher education	11	38%	21	84%
**Household net income (prior to corona), net income**				
Up to 1400€ (1200GBP)/month	4	14%	2	8%
1401(1201)–3000€ (2600GBP)/month	19	65%	6	24%
More than 3000€ (2600GBP)/month	6	21%	17	68%

One author (Galasso) went through the whole Irish and Italian dataset to ensure a comprehensive understanding. For this paper, the data code ‘about children general/specific’ and ‘about youngster general/specific’ were analyzed. This second code is relevant to this paper as, although we always write here about ‘children’, our focus is school aged children, and this includes teenagers who, in the interviews, were sometimes referred to as ‘youngster’ rather than ‘children’. In T2, another relevant code–‘school’–was added. The Italian quotes used in this paper were translated into English by a native Italian speaker (one author) and checked for accuracy by a native English speaker (the other author).

## Results

Several of our participants engaged with the public health dilemma around schools, by displaying their positions on whether to prioritize school opening or not for the containment of the pandemic. Most of them recognized the importance of children being able to access education as vital on many levels:

‘Education is the basis for everything. Knowledge is also a proper growth for the child, at social level. It is important. It is a second family. If educational bases are missing, the outcomes cannot be positive’ (IT-T2).

However, the opinions of participants varied considerably as to whether measures to contain the pandemic and therefore reduce the potential number of deaths, measures such as school closures were justified. According to some, the containment of the pandemic was to be prioritized over schools:

‘I think that the whole psychological and social issue of children that need to go to school is of minor importance than a potential increasement of deaths’ (IT-T2).

On the opposite, other participants argued that schools reopening was an ‘acceptable risk’, that it was an okay trade-off that numbers of cases would increase. Although they agreed that ‘sure a few more people could die’, they believed that the need to educate children in a physical school setting overrode the need of the public’s health: ‘life has to go on’ (IE-T2).

In addition to their general opinions, participants often reported their lived experiences and impressions related to the implementation of both options (schools closed and schools open), by articulating a variety of issues and challenges that highlight inequalities in different contexts that are the main focus of this paper: (1) *Inequalities in access to education during school closures*; (2) *Inequalities in the home setting during school closures*; (3) *Inequalities in school preparedness to reopen.*

(1) *Inequalities in access to education during school closures*

Most participants whose children experienced distance learning were extremely critical and considered it inadequate and insufficient compared to what children could have learnt in school. However, substantial differences emerged in the ways distance learning was implemented and perceived.

Overall, many participants believed that school closures had been for children ‘a terrible interruption in their education’ that was ‘only going to impact their development’ (IE-T2). On the other hand, some participants did reflect positively about children being at home more, arguing that they were ‘going to learn far more in life skills than they ever will in the academic setting’. (IE-T1). The difference in the perspective of our respondents in this regard, appeared to be crucially dependent on whether or not someone within the family setting had the capacity to oversee the education of these life skills.

Interview respondents also described unequal ability of schools to adapt during this period of closures, and subsequent inequalities in the education received by children during this time. Some participants declared themselves quite satisfied as schools and teachers reacted promptly to the new situation: ‘the teachers have always been helpful, so no problem’ (IT-T1). Whereas other participants complained that they did not receive any help in accessing teachers and education: ‘the teachers did not let themselves be seen or heard, never’ (IT-T1). Some schools arranged a very structured online timetabled approach: ‘it is really like if they were actually at school’ (IT-T1), while other participants complained of the opposite, that they were left structureless:

‘a teenager is going to tell you: “who cares, there is no school bell, nobody is waiting for me, what is the problem if I wake up at 8? I can catch up later if there is no scheduled class’” (IT-T1).

Also, participants reported different experiences around the assistance that they or other adults could provide to children for their education. Some parents explicitly realized that they had a new role in their children’s education: ‘now I have to be the teacher’ (IE-T1). Most reported that for them it was ‘quite a challenge actually to do home-schooling’ (IE-T1), whereas some parents in lockdown were actual teachers, so they just adopted a new norm whereby they became their children’s teacher:

‘I have neighbours where both parents are teachers and it is like a religion nearly every morning the kids have to be up, do work, do the school programme that comes on RTÉ^1^, that 11 to 12 or something, that has to be done. Like the morning of every day is set out for school and then the afternoons are free’ (IE-T1).

Others, on the contrary, emerged as completely unable to help their children and even to provide access to technology for educational purposes within the home setting:

‘sometimes some children lose the connections, other mothers at the other end of the call are in difficulty as well […] some do not even have the tools, they have no equipment or no internet connection’ (IT-T2).

Although all the parents were implicitly requested to become their children’s teachers, the interviews evidenced that not everyone had the same competences, capacities and equipment to successfully comply with this role: reported or emerged inequalities in family situations, as well as inequalities in schools organization and in teachers helpfulness, appeared to correspond to very unequal educational settings during school closure.

(2) *Inequalities in the home setting during school closures*

Different–or unequal–capacities of family support were not reported only in the context of homeschooling, but around all the aspects of children’s lockdown. Importantly, the situations narrated in our interviews remarked that not only children, but also their families were exposed to unequal challenges: if school closure disrupted children’s education, in some cases it also appeared as disrupting the family life and home setting, and in some cases even parents’ working life–with further reverberations on children.

While many participants expressed concern and sorrow for children not going anywhere, just being at home and missing their friends and their lives, to the extent that they claimed that ‘Children are the primary victims of all this situation’ (IT-T1), different home settings emerged as possibly alleviating or worsening the situation.

Some participants described their efforts to lighten the mental burden on their children, like allowing some minor infringements to the restrictions to let them go out or see their friends:

‘We obey most of the rules most of the time and I think it is a matter of balance and sometimes it can be you know we have our children’s mental health to consider’ (IE-T2).

Others reported their efforts in communicating the pandemic situation to children, to achieve a good balance between letting them understand the seriousness of COVID-19 without exacerbating anxiety:

‘The news, some can be rather tough. We, as adults, have followed this news. Then we have reported this to the children to let them understand that the situation was serious, but without creating too much tension and anxiety’ (IT-T1).

Some participants committed to providing their children with ‘the same kind of environment that you had before, but obviously under completely different conditions…’ and by finding a new routine that ‘especially for the kids that they have a sense of it’s all normal, this is our new norm, it’s fun, you know there’s nothing to worry about’. (IE-T1).

On the opposite, participants expressed major concern for extreme situations, such as ‘situations of violent or alcohol addicted parents’ (IT-T1), or children in the context of domestic violence or abuse:

‘…there are housing issues here in Ireland anyway, so you could have overcrowded houses, you could have houses full of kids that are just very difficult, you could have domestic violence, the obvious issues, all those issues I think are really stretching everyone at the moment’ (IE-T1).

These remarks importantly recalled that, if some children had parents who improved their situations by mediating information for them and committing themselves to building the children a routine as normal as possible, in some cases parents in abusive environments only worsened the situation for their children.

From their part, parents also reported challenges they faced themselves by reorganizing the routine and the spaces in the house to accommodate children staying home rather than at school. Also in this respect, very different scenarios were described, with some participants even reporting some positive aspects for the children as well as for themselves:

‘the kids are kind of playing around and doing stuff like playing cards and having quizzes that we never did before, so it is not all doom and gloom, for me’ (IE-T1).

An important difference was described as relating to having someone in the household who was not working:

‘At home obviously my wife is at home with the children all the time, but she would stay at home anyway so she would have been at home with them, so she hasn’t had to give up work or anything like that. So the impact from that perspective is quite limited’ (IE-T1).

Conversely, working participants and participants in the job market reported their situation as very challenging: some participants working from home with children were struggling to make rearrangements ‘so splitting up the days’ between working and looking after the children (IE-T1). In the interviews, major concern was expressed around parents who were not allowed to work from home when schools were closed, as it was a widespread case in Italy where, at the time of the T1 interviews, most workplaces were about to reopen, while schools remained closed. This was also mentioned as a reason to reopen schools:

‘Schools are important, because if you don’t know what to do with your children, there is no way you can go back to work’ (IT-T1).

This narrative was exacerbated in T2 when talking about the potential for further school closures:

‘I have some anxiety in this respect: anxiety not to find a job before things go really bad, and anxiety to do find a job before things go really bad. Maybe schools will close again, and I find myself like many friends of mine during lockdown, two parents working full time from home and having children to watch, because you cannot send them to the grandparents or to school. I am scared about both alternatives, neither is good’ (IT-T2).

In the regions where school closure was already happening again, T2 participants complained that:

‘the situation for working women is very tough […] The other time we were in lockdown and the mother was at home as well, so there was no problem. This time, with this partial closure, working women have to go to work while children have to stay home’ (IT-T1).

The interviews remarked how school closure had a major impact not only on children’s life, but also on their parents’ and carers’ life: an impact bringing unequal consequences to the home setting and to children themselves, which emerged as importantly related to parents’ working position, including the flexibility they were allowed or not allowed in terms of working times and remote working.

(3) *Inequalities in school preparedness to reopen*

When interviewed after schools reopened, participants reported different experiences upon schools’ organization and preparedness to reopen in time of a pandemic.

Some reported that schools were ‘well organized since the first day’ (IT-T2), and of school principals who ‘have put a lot of effort in the background to get everything up and running to be as safe as possible and as comfortable as possible for the kids’ (IE-T2).

Opposite to this, some Italian participants complained that schools reopened but teachers were absent, and the time schedule was not met: ‘Some teachers are missing. I don’t know exactly why….I even stopped asking. Classes are not going well’ (IT-T2). As a similar experience, another participant reported of a family member working as deputy principal in a school, who was overstressed because they did not have enough teachers:

‘Teachers are staying at home and I don’t have anyone who can substitute them….I don’t know what to do…if five or six teachers stay at home I can find a solution, but if next week five more teachers are missing, what do I do? There are no teachers and no substitutes’ (IT-T2).

Participants also commented upon safety measures implemented within schools. They were generally positive about them, although some participants considered some specific measures ‘too strict’, or even senseless, to the detriment of children’s well-being: for example, a participant working in a school commented that the requirement for children not to move from their seats during breaks as ‘an atrocity’, as it is believed that contagion outdoor is unlikely to happen. As a strategy to save well-being together with safety, this participant commented upon the benefits of transparent facemasks, as ‘it allows children to see the smile of the teacher’ (IT-T2).

On the other hand, participants expressed major concerns for children’s exposure after the school day has finished, that according to some was ‘undoing all the hard work’ (IE-T2). In that respect, the major concern among Italian respondents was public transport that children had to take to go to school:

‘We do all that, we are super careful, staggered entrances and exits, and then - fantastic – overcrowded buses! What did we go to school half an hour in advance for, if then the bus is overcrowded and there is no distancing on the bus?’ (IT-T2).

This unequal preparedness of schools and school transportation emerged out of the interviews as impacting the returning school children differently. Our respondents described critical differences in schools’ ability, management and resources, that we noticed corresponded to different–and unequal–levels of education delivered to children upon their return to school.

## Discussion

Participants’ displayed positions around the issue whether to keep schools open for the sake of children’s education and well-being, or closed for limiting chances of contagion across the population, reinforced our intuition that neither of these moral requirements unproblematically overrides the other, and that their conflict is a genuine public health dilemma. As a matter of fact, participants claiming that one moral requirement overrides the other disagreed on which was the overriding one, and many participants displayed mixed feelings rather than taking an absolute position for one or the other.

What is most important about our data, however, is not that they support the genuineness of the dilemma, but that they indicate how, in either way, the dilemma generates different and unequal consequences to different people: a wide range of consequences spanning from manageable challenges even associated to some benefits for some (like in the case of the participants reporting their engagement with their children with family activities and games), to major and possibly long-lasting disruption for whole families for others (like in the case of the participants reporting to be affected in their job or job search).

The inequalities and inequities that emerged out of the interviews associated with the two dilemma options (schools open versus schools closed) do not help to solve the public health dilemma by indicating that an option is morally preferable to the other, and indeed it was not the aim of this paper. Both options of the dilemma as described by our interviewees are actually associated with unequal challenges: exacerbation of existing inequalities is disgracefully associated with several aspects both of the pandemic itself and of its containment measures (Fiske et al., 2021). As a consequence, rather than arguing in favour or against containment measures, such as school closures, in relation to the inequalities that they generate or exacerbate (Kraaijeveld, 2021), we claim that institutions should take these inequalities into account and strive to mitigate them regardless of which option or strategy they adopt to harness the pandemic.

Informed by the specific challenges we learned from the interviews, we encourage normalizing the consultation of concerned subjects in relation to moral or public health dilemmas. As we claim that, especially when decisions around a public health dilemma are taken in conditions of uncertainty (Hansson, 2013), taking into account the full range of consequences in either scenario, including the specific disproportional challenges affecting some, would provide a more comprehensive picture of what is at stake with either option and help with the decision accordingly. Moreover, we argue that, while a dilemma cannot by definition be solved, knowing the specific challenges the two conflicting options raise can help to mitigate their detrimental consequences (Weinstock, 2020). Our participants reported a variety of experiences and impressions related to both the scenarios associated with the dilemma, which show how different contexts and infrastructures minimized or amplified the damage to children’s education and well-being. Importantly, the reported differences highlight actionability on some of the damages. At least in some cases, the fact itself that these differences exist is a demonstration that a different scenario is possible: the most positive reported experiences to demonstrate that it is possible to mitigate harms at least to that extent.

### Inequalities ‘to the Greatest Burden of the Least-Advantaged Members of Society’

The political philosopher John Rawls, when articulating his influential Difference Principle (Rawls, 1971), famously argued that for fair distributive justice ‘social and economic inequalities […] are to be to the greatest benefit of the least-advantaged members of society’ (Rawls, 2001: 42–43). Opposite to this, and thence contrary to the (Rawlsian) principles of distributive justice (as well as contrary to any egalitarian approach), the inequalities emerged out of our interviews are ascribed to conditions that we observe critically relate to pre-existing disadvantages: thence we argue that the burdens associated to closures and reopenings of schools in the context of the COVID-19 pandemic disproportionally weighed on those already socioeconomically and educationally least-advantaged, to the further detriment of social justice.

Overall participants agreed that with school closure children’s education was drastically compromised, and that distance learning provided by schools, as well as the assistance provided by parents, could not fully compensate for in person lectures. However, in some cases the situation was described as much worse than in others: children that for whatever reasons could not count on full family support, and children whose schools and teachers were less able to adapt, less equipped and not as proactive, are those who emerged as getting the most detrimental effects in terms of education during lockdown. While some participants reported of children being abandoned by schools and of parents unable to help them in their educational needs, other participants reported that some more organized schools, and parents who had the capacity—such as parents not working or with more flexible working time and/or having competences such as more (tech-) literacy or parents who were actually teachers—managed to provide children with better educational support during school closures.

From the scenarios described in the interviews we observed that, while for some families having children at home was nothing but a challenge that they managed to deal with, even with some positive results, for others it was a desperate situation: the lack of possibility of working from home or of flexible working times for some, caused in some cases very tough situations, once again to the detriment of the concerned children. The impression from the information we collected, complemented with the material from the other studies and reports referenced throughout this paper, is that the families most negatively affected by this public health dilemma are likely to be those already socioeconomically disadvantaged: parents who cannot afford not to be working, or parents in lower employment grades who are less able to negotiate flexible working conditions.

Similarly, in terms of mental and social well-being, our participants expressed major concern for all children in relation to isolation and impossibility to see their friends and extended family members. However, some participants reported family efforts to help children deal with the situation by mediating information to minimize trauma and feelings of uncertainty, by recreating an environment of apparent normality and even fun in the house. Conversely, major concern was expressed about abusive home settings and of domestic violence. Also in this respect, although mental health consequences are seriously concerning for every child, the interviews described important inequalities, related to and exacerbating pre-existing disparities.

Also in relation to schools opening our data showed unequal and potentially actionable challenges. Concerning the safety in schools in preventing the children catching or spreading COVID-19, many participants were satisfied with the measures adopted. However, some participants complained that measures were abandoned as soon as the children left the school premises, and particularly on public transport, thereby ‘undoing all the hard work’ (IE-T2). Participants also reported different degrees of organization in different schools, which compromised good education deliveries in certain schools.

The interviews illuminated how both keeping schools open and keeping schools closed had incommensurable devastating effects on children as well as on population well-being and health. However, respondents reported important differences that we argue in most cases exacerbated pre-existing inequalities and inadequacies: most likely, if parents lack capacity or skills to assist their children’s learning or to provide them with recreational activities during school closures, or if a home setting lacked the infrastructures to secure children’s well-being and educational tools during the pandemic, these capacities, skills or infrastructures were also lacking before the pandemic—but importantly they were at least to some extent possibly compensated in the school setting. Conversely, if some schools lacked organization, proactive and helpful teachers and infrastructural support during the pandemic, they were most likely wanting in these aspects also before and beyond the pandemic—although the pandemic undoubtfully added more challenges to the previous ones.

### Potential Actions to Tackle Educational Inequalities during and beyond a Pandemic

Addressing the inequalities discussed above can mitigate the effects of the public health dilemma in relation to school closure-reopening and reduce the harm in either case, and can also reduce inequity beyond the context of the pandemic (Fiske et al., 2021).

Not all the emerged inequalities are actually actionable, at least in the short term: flexible working arrangements can possibly improve parents’ capacities, but if for example lack of skills (and/or of readiness or willingness) of some parents to assist their children education are also at stake, this is notoriously not a problem that the implementation of a policy can solve unproblematically overnight (see Fishkin, 1983; Freeman, 2007; Brighouse and Swift, 2009). On the other hand, sometimes the reported lived experiences of participants themselves indicated potential strategies to mitigate the damage. In the case of school closures, the described challenges suggest that some actions could be taken to facilitate support from schools and from families to assist children’s education and well-being, at least to a certain extent. For example, our interviews suggest that flexible working arrangements for parents, and in some cases financial support, could at least in some settings alleviate pressure on parents and allow them adequate time and energy to assist in their children’s education and to provide them with entertainment, as some parents actually were reported to have done: consistently, we recommend that governments encourage and facilitate companies to allow some flexibility to working parents, at least (although possibly not only) during public health emergencies. The challenges reported by some participants about dealing with children’s trauma and uncertainty, suggest that access to external consultants and experts could be a vital resource to provide critical help to parents in this regard. The successful achievements as reported by our respondents around some schools suggest that better equipped and adaptable schools could provide appropriately structured and coherent distance learning, and we recommend that governmental support in this direction may thence minimize the harm in terms of education loss.

In the case of schools opening, positive experiences reported by our interview participants suggest that action could be taken to minimize the risk of contagion without exacerbating children’s discomfort in relation to restrictions. On the one hand, the interviewees stressed the importance of schools having the necessary arrangements in place to ensure that there are enough teachers to cover the whole school timetable, to the benefit of education adequacy. On the other hand, respondents suggested how some simple expedients would make measures and restrictions more tolerable to children without impacting on contagion risk: for example, respondents suggested that teachers could wear transparent facemasks so that their facial expressions and mouth movements can be seen by the children; that children could spend their school breaks outdoors rather than in the classroom, or there could be recreational activities organized by the school during the school day that do not add further risk with regards to spreading the virus.

We are aware that it is not always so simple, indeed quite the contrary, we do acknowledge that in most cases inequalities and challenges are related to upstream, complex, structural inadequacies, involving both families and schools (Creary, 2021). The strategies listed above can mitigate some short terms effects of the current pandemic on children, especially in not-extreme situations. However, for cases of domestic violence or abuse, or of total lack of family support, or for schools unequipped and unable to assist children especially from problematic socioeconomic backgrounds or with special needs, more radical and long-term strategies, social reforms and financial plans need to be implemented (OECD, 2020; United Nations, 2020; Creary, 2021). Importantly some of these inadequacies, and some other minor ones reported in our interviews, are not derived from the pandemic: the COVID-19 pandemic, rather than creating new ones, evidenced long-lasting structural inequalities and inadequacies affecting children and schools (Doyle, 2020; Marmot et al., 2020; OECD, 2020; UNESCO, 2022). In that respect, the pandemic can and should be regarded as an opportunity to detect and to tackle pre-existing structural inequalities regarding schools and society in general (Fiske et al., 2021).

The lived experiences reported by participants played a central role in the identification of specific challenges and inequalities to be tackled. Our participants provided a helpful first overview of what did not work properly and could be improved. We conclude that consultation of concerned actors is a vital mechanism for identifying inequalities and inadequacies that can be potentially overcome (Callon et al., 2009), and we therefore suggest public consultation to be implemented at a deeper and larger scale, particularly in the context of inadequacies and inequalities around children’s education and well-being, through and beyond the COVID-19 pandemic. For example, we believe it would be very useful to implement nationally organized qualitative interviews in every school on a regular basis with teachers, school staff, parents and, importantly, children. This would help to collect first-hand lived experience that can illuminate the needs, the inadequacies and disparities requiring deeper commitment or concrete action from the school and/or from the State.

## Conclusion

In this paper, we argued that, although to sacrifice children’s education and well-being by closing schools, as well as to sacrifice the safety of the population by not constraining the spread of the pandemic when needed, are both not acceptable options, some of the damages caused by either option can be mitigated by adequate policies and measures (Weinstock, 2020). Our interviews in Ireland and Italy highlighted some unequal and potentially actionable challenges associated to both scenarios, and we suggest that, although underlying structural inequalities require radical and long-lasting social reforms, the reported lived experiences of the directly concerned public could provide some helpful first guidance to policymakers to overcome some inadequacies and minimize damages.

On a broader perspective, we claim that effective consultation of the concerned actors could provide informative guidance in the context of any public health dilemma: potentially affected people, by voicing their specific challenge, could provide public health authorities with a better understanding of the real concrete implications of the dilemma, and can—like in the case we discussed here—help address strategies to minimize the adverse consequences of either option.

## Footnotes

1 RTÉ is the Irish public service broadcaster—Raidió Teilifís Éireann (RTÉ) which showed every weekday a one hour children’s educational programme whilst schools were closed.
